# Ebola virus disease in the eyes of a rural, agrarian community in Western Nigeria: a mixed method study

**DOI:** 10.1186/s12889-020-09441-7

**Published:** 2020-08-31

**Authors:** Abiodun Benjamin Idowu, Ifeoma Peace Okafor, Ezekiel Sofela Oridota, Tochi Joy Okwor

**Affiliations:** 1 Department of Community Health & Primary Care, College of Medicine, University of Lagos, Lagos, Nigeria; 2 Nigeria Centre for Disease Control, Abuja, Nigeria

**Keywords:** Ebola, Infectious disease, Epidemic, Rural, Knowledge, Attitude, Nigeria

## Abstract

**Background:**

Ebola virus disease (EVD) is a severe hemorrhagic disease caused by Ebola virus. Several outbreaks have been reported in Africa and often originated from remote agrarian communities where there are enormous misconceptions of the disease, refusal of early isolation and quarantine, and unsafe burial rites practices which aggravates the epidemics. It is on this basis that this study was conducted to (assess) the knowledge, perceptions, beliefs and preventive practices against EVD in a predominantly agrarian rural community in Southwest Nigeria.

**Methods:**

This was a cross-sectional study conducted in Igbogila town, Yewa North Local Government Area of Ogun State, Southwest Nigeria in the latter part of 2014 during the EVD outbreak. Mixed methods were used for data collection. Quantitative data collection was done using a pre-tested interviewer administered questionnaire. Four hundred and seven respondents selected by multi-stage sampling technique were interviewed. Descriptive and inferential statistics were done, and the level of significance was set at 0.05. Qualitative data collection involved four focus group discussions a year after the epidemic was declared over in the country. The discussions were recorded, transcribed and analyzed along major themes.

**Results:**

All respondents were aware of EVD with radio and television being the major sources of information. Knowledge of the disease was however very poor with many misconceptions and it was significantly influenced by educational level of respondent. EVD survivors will be welcomed back into the community by few residents (36.8%) and a much fewer proportion (27.2%) will freely entertain a survivor in their house. Most would prefer local herbalists over orthodox medical practitioners to care for their loved one in case they contract EVD. Although respondents knew that burying a victim is dangerous, they opposed cremation.

**Conclusion:**

There was poor knowledge of EVD with a lot of misconceptions. Community members were not pro-active about prevention with dire consequences in the event of an outbreak. Continuous public education should be done via mass media, traditional institutions and other community-based channels as part of emergency preparedness.

## Introduction

Ebola Virus Disease (EVD) is a severe hemorrhagic disease caused by Ebola virus: a non-segmented, enveloped, negative-strand RNA virus [1]. The first case of EVD was identified in 1976, since then, several outbreaks have been reported in Africa [2]. In the last 20 years, an outbreak of EVD has been reported at least every 3 years [3, 4]. In 2014; the deadliest, most widespread (affected ten countries), EVD outbreak that lasted approximately 2 years occurred making it a global emergency [5]. Current Corona Virus Disease (COVID-19) pandemic has again brought to the fore, the need for countries to maintain a high standard of preventive measures and preparation for emergency response for any emerging or reemerging infectious disease. Populace needs to be enlightened on EVD preventive measures such as maintenance of careful hygiene (washing hands with soap and water or an alcohol-based hand rub), avoiding contact with non-human primates and bats, avoiding contact with infected person’s body fluids or infected items, and avoiding funeral or burial rituals that require handling of the body of someone who has died from EVD (confirmed or suspected) [6].

Public health response to EVD outbreak include: case finding (suspected, probable and confirmed), contact tracing, isolation and early quarantine, treatment of symptomatic cases, and ensuring appropriate burial for the deceased [7]. However, a closer look at past EVD outbreaks revealed that they often originated from rural agrarian communities where there are many misconceptions about the disease, refusal of early isolation and quarantine, and unsafe burial rites practices which aggravate epidemics [8, 9]. It is on this basis that this study was conducted to assess the knowledge, perception, beliefs and preventive practices among residents of an agrarian community in Ogun State, Southwest Nigeria. Findings will provide useful information to aid future outbreak prevention and control as well as emergency preparedness efforts.

## Methods

This was a descriptive cross-sectional study which employed mixed-method (quantitative and qualitative) approach in data collection. The study setting was Igbogila town, Ibeshe Ward, Yewa North Local Government Area of Ogun state, Southwest Nigeria. Yewa North is located at the West end of Ogun State sharing border with Benin Republic (a neighboring country). Igbogila is predominantly rural and agrarian with many of the residents engaged in agro-forestry related occupations. At the time of the study, the town had one Primary Healthcare Centre, two public secondary schools, five public primary schools, one major market, few churches and mosques. Only residents between 18 and 70 years that had been living in the study area for at least 6 months prior to the study participated in this study.

Quantitative data were collected during the Ebola epidemic in Nigeria (July – September 2014). Sample size was determined using Cochran’s formula (*n* = z^2^p(1-p)/e^2^) [10]. The calculation was based on: prevalence of good knowledge (p) of 53% obtained from a similar study [11], standard normal deviate (z) at 95% confidence being 1.96 and 5% margin of error (e) resulting in a minimum sample size of 383. This was increased by 10% (38) to make up for non-responses and incomplete questionnaires giving a total sample size of 421.

Multi-stage sampling was used to select the respondents. In the first stage, one ward (Ibeshe) was selected from the eleven wards in Yewa North using simple random sampling technique (balloting). In the second stage, one town (Igbogila) was selected from the seven towns in Ibeshe ward. Igbogila comprises nine smaller communities which were all included in the study. Respondents were equally allocated to the communities i.e. about 47 respondents were required from each community. The third stage involved the selection of houses following enumeration and systematic sampling of houses. The houses in the communities largely had no numbering system, so, the research team carried out house numbering. In the fourth stage, households were selected from the houses. Only one household was selected per house (simple random sampling (balloting) was used to select one when there were more than one household). In the final (fifth) stage, respondents were selected from households. Only one respondent that met the inclusion criteria was interviewed per selected household (simple random sampling (balloting) was used to select only one respondent when there were more than one eligible respondent).

Respondents were interviewed face-to-face using a pre-tested interviewer administered questionnaire adapted from similar studies [11, 12]. Eight research assistants who were fluent in Yoruba, English and ‘Pidgin’ English were trained for data collection. The questionnaire sought information on socio-demographic characteristics, awareness, knowledge, attitude and perception of EVD. Knowledge was assessed using questions on cause, transmission, symptoms, prevention and cure of EVD. Perception and attitude to EVD were assessed using respondents’ agreement or disagreement to a set of Likert Statements. Data were coded, entered and analyzed using Epi Info™ 7.0 statistical package [13]. Descriptive statistics (frequency, mean and standard deviation) and inferential statistics (Chi-square test) was used to test association between categorical variables. Level of significance was set at 5%.

In the knowledge section, each correct response given by respondents was allotted one point. Overall knowledge was assessed using five domains: cause (1 point), transmission (3 points), symptoms (5 points), prevention (5 points) and cure (1 point). This gives a total maximum score of 15 points converted to percentage. Using 50% cut-off point; respondents with total score < 50% were graded as ‘poor knowledge’ while those with > 50% were graded as ‘good knowledge’. Attitude was scored using three points Likert scale; the maximum obtainable score was 21 and the least was 7. Using the mid-point (14) as cut-off point, respondents with score < 14 were graded as having “poor attitude” while those with scores > 14 were graded as having “good attitude”.

For the qualitative aspect, focus group discussions (FGDs) were conducted in November 2015, about a year after the epidemic was declared over by WHO [14]. The main purpose for the FGDs was to explore explanatory models for the disease in rural communities and their preventive practices against an outbreak. According to the WHO, this is important in any epidemic preparedness and response [15]. Discussants were approached face-to-face and selected into one of four groups: higher secondary education students (7 discussants), females of reproductive age (6 discussants), adult male (6 discussants), elderly female (6 discussants). FGD participants were selected by purposive sampling as discussants in each group were selected to be of the same gender and about same age as suggested by Ritchie and Lewis qualitative research framework [16]. In each group, one of the discussants volunteered his/her home for the discussion. FGDs were moderated by the principal researcher with the assistance of one note taker and a time-keeper. Each session lasted for about 2 h. Discussions were held mainly in local (Yoruba) language understood by all the participants and tape-recorded in addition to notes. Each discussant was assigned a number. At the end of each session, discussants were given light refreshments. The recordings were later translated and transcribed in English. Data was saturated in domains of cause, and spread of EVD, but, unsaturated in domains of treatment. Thematic analysis was done manually – recurring themes from the data were identified, emerging patterns noted, and report written based on these identified patterns. For the purpose of presentation, the groups were coded as follows: Higher Secondary School students (HS), Adult males (AM), Older females (OF), and Women of reproductive age (RF).

Participation was voluntary and formal consent was obtained from each participant. Respondents were informed of their right to withdraw at any point of the study without prejudice in line with Helsinki declaration [17].

## Results

### Results of quantitative study

A total of 407 respondents completed their interviews while 14 respondents withdrew their participation, making the response rate to be 96.7%. Mean age of respondents was 34.8 + 12.8 years with slightly more males (*n* = 221, 54.3%). Almost one-quarter, 97 (23.8%) had no formal education and half (*n* = 204) earning less than 18,000 Naira monthly (less than 50 US dollars) (Table 1).

**Table 1:** Sociodemographic characteristics of respondents

Variable (***N*** = 407)	Frequency (%)
**Age (years)**	
18–35	240 (59)
36–50	113 (28)
51–70	54 (13)
Mean 34.8 + 12.8 years	
**Sex**	
Female	186 (45.7)
Male	221 (54.3)
**Marital status**	
Married	236 (58.0)
Never married	131 (32.2)
Divorced/widowed/separated	40 (9.8)
**Education**	
No formal education	97 (18.4)
Primary	86 (21.1)
Secondary	149 (36.6)
Tertiary	75 (18.4)
**Monthly income (Naira)**	
< 18,000	204 (50.1)
> 18,000	203 (49.9)

### Awareness and knowledge of Ebola virus disease

All the respondents were aware of EVD. Majority, 281 (72.5%) heard of it through radio and television, 60 (15.5%) got to know from their friends or family members. Print media such as newspapers and flyers were not reported as a source of information by the respondents. ‘Dirty environment’ was the most commonly mentioned cause of EVD (34.4%), only 64 (15.7%) knew that a virus is implicated. Few (39.1, 36.9 and 24.1% respectively) knew that eating poorly cooked bush meat or contact with non-human primates or contact with body fluids of infected persons pose risk of EVD transmission. A minority knew fever (34.9%), vomiting (29%) and headache (28%) as symptoms of EVD. (Table 2).

**Table 2:** Knowledge of cause and route of transmission of EVD

Variable	Frequency (%)
**Cause of EVD** (*n* = 407)	
Dirty environment	140 (34.4)
Germs (Virus)	64 (15.7)
Our sins	24 (6.0)
Bush meats	21 (5.1)
Witches and wizards	13 (3.1)
Curses	11 (2.8)
Don’t know	134 (32.9)
**Means of transmission of EVD** (multiple responses)	
By eating improperly cooked bush meat	159 (39.1)
By direct contact with non-human primates e.g. monkeys, bats	150 (36.9)
Through unprotected sexual intercourse with an infected person	111 (27.3)
By direct contact with blood and body fluids of infected person	98 (24.1)
Through mosquito bite	65 (16.0)
By direct contact with asymptomatic carrier	60 (14.7)
By direct contact with infected objects	54 (13.3)
Through air	41 (10.1)
By eating food from canteen	41 (10.1)
**Symptoms of EVD** (multiple responses)	
Fever	142 (34.9)
Vomiting	118 (29.0)
Headache	114 (28.0)
Body weakness	83 (20.4)
Diarrhea	80 (19.7)
Body rashes	73 (17.9)
Joint and muscle ache	71 (17.4)
Internal / external bleeding	71 (17.4)
Sore throat	62 (15.2)
Abdominal pain	62 (15.2)
Don’t know	151 (37.1)

A third (33.4%) knew hand washing and avoidance of contact with non-human primates as preventive measures while only 37 (9.1%) knew that avoiding funeral or burial rituals involving contact with victims’ corpses is a preventive measure (Table 3).

**Table 3:** Knowledge of prevention and cure of EVD among the respondents

Knowledge of EVD	Frequency (%)
**Preventive measures against EVD** (multiple responses)	
Avoiding direct contact with people	160 (39.3)
Washing hands frequently with soap and water	136 (33.4)
Avoiding contact with non-human primates’ blood and fluids	104 (25.6)
Avoiding contact with items that have come in contact with infected persons	101 (24.8)
Bathing with salt and hot water	60 (14.7)
Ensuring proper environmental and personal hygiene	58 (14.3)
Washing fruits and vegetables thoroughly before consumption	58 (14.3)
Seeking medical care immediately (on suspicion of) EVD symptoms	57 (14.0)
By using hand sanitizer	45 (11.1)
Avoiding funeral rites requiring handling people that died of EVD	37 (9.1)
Don’t know	86 (21.1)
**Cure of EVD**	
There is yet no drug that can treat EVD	128 (31.4)
EVD can be cured with antibiotics	51 (28.5)
Local herbs and concoctions can treat EVD successfully	40 (12.5)
Traditional healers can treat EVD successfully	33 (9.8)
Spiritually healers can cure EVD successfully	20 (8.1)
Medical doctors have a definite drug that can cure EVD	19 (4.9)
Don’t know	116 (28.5)
**Overall knowledge of EVD**	
Good knowledge	30 (7.4)
Poor knowledge	377 (92.6)

Neither age nor sex of the respondents significantly influenced their knowledge of EVD. However, those respondents with at least secondary education were more likely to have good knowledge of EVD (*p* = 0.002) (Table 4).

**Table 4:** Factors affecting knowledge of EVD

Variable	Knowledge of EVD				
	Good(%)	Poor(%)	Total	X^**2**^	***p***-value
**Age group**					
< 35	20 (8.3)	220 (91.7)	240		
> 35	10 (6.0)	157 (94.0)	167	0.79	0.485
**Sex**					
Female	9 (4.8)	177 (95.2)	186		
Male	21 (9.5)	200 (90.5)	221	3.22	0.073
**Education**					
Secondary and tertiary	25 (11.2)	199 (88.8)	224		
Primary and no formal education	5 (2.7)	178 (97.3)	183	10.48	**0.002**

### Perceptions and attitude to EVD

A majority (82.1%) believed that EVD really exists and 313 (81.3%) perceived it to be very fatal. While 173 (45.2%) agreed that it is not curable, 240 (62.2%) believed that victims could survive if given prompt medical intervention. Almost a quarter, 89 (23.1%) saw it as a political ploy that government officials wanted to use to embezzle funds and only about half 199 (51.6%) thought that the country was truly Ebola free (Table 5).

**Table 5:** Perceptions/beliefs about Ebola Virus Disease

Statements	Agree(%)	Neutral(%)	Disagree(%)
EVD truly exists	316 (82.1)	34 (8.8)	35 (9.1)
EVD is not a ploy of the whites against African Countries	228 (59.1)	70 (18.1)	85 (22.0)
EVD is a fast-killer disease	313 (81.3)	48 (12.5)	24 (6.2)
EVD victims has a higher chance of survival if taken immediately to hospital for treatment	240 (62.2)	70 (18.1)	76 (19.7)
EVD is a ploy of the government and health officials to embezzle money	89 (23.1)	109 (28.2)	188 (48.7)
Someone who contracted Ebola disease reduces the chance of infecting others by going to hospital	248 (64.2)	71 (18.4)	67 (17.4)
EVD cannot be easily cured	173 (45.2)	105 (27.4)	105 (27.4)
Nigeria is truly EVD free	199 (51.6)	109 (28.2)	89 (23.1)

In respect to their attitude; 301(78%) reported that they would accept to be quarantined if they were found to have had close contact with a case, 255(66.1%) would support and empathize with a friend or relative who is infected, however, only two-fifths 162(42%) would buy from a shopkeeper who has recovered from EVD and even a lesser proportion 142(36.8%) would welcome a survivor back to the neighbor-hood. Only 105(27.2%) would entertain a survivor in their homes. Overall, respondents 357(88%) had a good attitude towards EVD (Table 6).

**Table 6:** Attitude towards EVD

**Statements**	**Agree(%)**	**Neutral(%)**	**Disagree(%)**
I try to avoid body contact as much as possible when I am in public transport	239 (61.9)	62 (16.1)	85 (22.0)
If I suspect Ebola symptoms or signs in my family member/ friend, I would voluntarily hand him/her over to appropriate health facility	275 (71.2)	39 (10.1)	72 (18.7)
If I am suspected to have come in contact with EVD case, I will accept to be quarantined	301 (78.0)	28 (7.2)	57 (14.8)
If I find out that a friend/ family member was infected with EVD, I would offer them support and empathize with them	255 (66.1)	41 (10.6)	90 (23.3)
I will buy from a shop-keeper who had contacted EVD but has recovered and declared well by medical doctors	162 (42.0)	70 (18.1)	154 (39.9)
I will welcome someone who has recovered from EVD back into the community or neighborhood	142 (36.8)	73 (18.9)	171 (44.3)
I will freely entertain someone who has recovered from EVD in my house	105 (27.2)	53 (13.7)	228 (59.1)
**Overall attitude grade**	**Good(%)**	**Poor(%)**	**Total(%)**
	357 (88)	50 (12)	407 (100)

## Results of FGDs

### Sociodemographic characteristics of discussants

The mean age of the FGD participants was 33 + 10.9 years, 15 (60%) were females, 10 (40%) had no formal education while 2 (8%) had tertiary education. The participants were largely farmers (36%), and petty traders (28%). One discussant was a herbalist (Table 7).

**Table 7:** Sociodemographic characteristics of FGD participants

Variable	Frequency (%)
**Age (years)**	
< 20	4 (16)
20–30	9 (36)
30–40	6 (24)
> 40	6 (24)
Mean 33.0 + 10.9 years	
**Sex**	
Female	15 (60)
Male	10 (40)
**Education**	
No formal	10 (40)
Primary	4 (16)
Secondary	9 (36)
Tertiary	2 (8)
**Occupation**	
Farmers	9 (36)
Petty trader	7 (28)
Students	6 (24)
Others^a^	3 (12)

^a^herbalist, teacher, bricklayer

### Transmission, prevention and treatment of EVD

The recurring themes on how Ebola disease can be contacted were: eating infected bush meat, unprotected contact with infected persons, and intercourse with multiple sexual partners.

“*It is gotten by coming in contact with infected animals, animals such as bats and bush meats*” –HS3 (19 years old male student).

“*The disease is catching whoever has sex with prostitutes …*” – AM6 (39 year old bricklayer).

More respondents believed that local herbalists know the cure for ailment.

“*I will call a herbalist to come and treat the person (a case) at home*” – RF (32 years old female farmer).

“*I believe that they are lying by saying there is no cure for the disease … … , if the victim is taken to good traditional healers, the person will be cured*” – HS6 (16 year old male student).

When asked how best to handle the corpse of a close relative that died of EVD, it was evident that the people knew that burying someone with EVD is not without any risk.

“*I will not move close to the corpse. The people who died of the disease are usually burnt but I cannot allow my own dead family member to be burnt. I will just call them at the centre (primary health facility in the area) to come and help me bury the corpse*” – HS3 (19 year old male student).

While some (8 out of the 25) of the respondents did not stop eating bush-meat (bats inclusive), many (12 out of 25) of the respondents stopped eating bush meat. The precautionary measure was however for a while as it was found that these respondents that initially restrained their intake of bush meat had resumed its consumption.

“*I stopped eating bush meat and bat, but when I later saw that people who ate bush meat did not die, I started eating them back*” – AM1 (38 year old male farmer).

The important theme that emerged on preventive measures for EVD was the use of salt water. Some respondents bathed with salt water, drank and mandated its use in their family till they experienced related adverse effects.

“*I bathed with salt water as instructed by my father*” – HS6 (16 years old male student).

“*My six children and myself used salt water to bath for some days but stopped when we started having skin rashes*” – OF3 (48 year old female trader).

Their current preventive practices were explored (without prompting). Majority of the respondents confessed that they were eating bush meat as before. They were mostly not taking any pro-active preventive measures to prevent EVD such as limiting close physical contact or direct contact with bush animals.

“*I am not doing anything. I am eating bush meat …*.” – RF (30 year old female farmer).

“*I am not doing anything … … I did not need to bother myself*” – OF2 (58 year old female).

## Discussion

At the outset of the EVD outbreak, the Nigerian government embarked on widespread health campaign with major attention on mass media. The mass-media platforms successfully raised EVD awareness as all the rural dwellers in this study were aware of EVD and they indicated that radio and television were their main sources of information. Mass-media played similar pivotal role in purveying awareness for residents of urban communities in Lagos, Nigeria [12] and for locals at epicenters in Sierra Leone [18].

However, the high level of awareness did not translate to better knowledge of the disease. Most respondents had poor knowledge riddled with many misconceptions. For instance, most of them either did not know the cause of EVD or misconceived the cause to be dirty environment. There are evidences that have implicated bush-meats especially non-human primates e.g. bats in the spread of EVD, yet only few (39%) knew that EVD is spread by contact with infected non-primate animals [19, 20]. The prominent misconception of the cause of EVD as revealed in the FGD was the belief that Ebola disease is acquired by leading a promiscuous lifestyle. This apparent disparity between biomedical and traditionally perceived etiology could stymie prevention in the event of another outbreak because based on etiological variances, local perception of prevention will conflict with orthodox suggestions [21].

Apart from the misconception of cause of EVD, the knowledge of community-based modes of transmission (from infected individual to others, and from infected fomites/objects to man) of EVD were also less known among the residents of the agrarian community. This is worrisome because during outbreaks, community-based transmissions are responsible for most secondary cases and thus responsible for perpetuating the spread of infection [22]. The knowledge of prevention of EVD was also found to be inadequate. More than 50% did not know that; avoiding direct contact with people, frequent hand washing, avoiding contact with non-human primates’ body fluids and blood, and avoiding contact with infected items are precautionary measures. When the respondents were asked how they will handle the corpse of a relative that died of EVD; it was evident that the people knew that burying someone with EVD is not without risk but they opposed cremation - “*… I cannot allow my own deceased family member to be burnt*”. Cremation is rejected because it is not culturally acceptable in most parts of West-Africa where autochthonous residents strongly believe that deceased soul will haunt living relatives if not given a traditionally acceptable burial [23]. This has potential to impede effective burial of dead cases and it can aggravate epidemics as evidenced by catastrophic events that followed unsafe burial of cases at the early stages of the 2014 outbreak (in Sierra Leone and Liberia) [24, 25]. It may be beneficial to gradually institute interventions involving anthropologists and traditional institutions to discuss and relay such messages at the grass root level.

Exploring the respondent’s knowledge of cure of EVD, it was found that although some (31.4%) knew that there is no cure for the disease, yet, most preferred local herbalists over orthodox medical practitioners to care for their loved one in case he/she contacts EVD. Being a rural setting, this is not surprising. The rationale behind this preference is the fear of having their relative isolated from them: “*… ..once they carry the person (victims) away from you, you will not be allowed to see them again …*” The discussants’ preference of local herbalist over medical practitioners is another cause for concern as such misconceptions had made people in Gulu district, Uganda to resort to traditional practices such as ‘*ryemo gemo’* rituals (wild shouting, jumping and running into Nile river), ‘*chani labolo’* rituals (slaughtering and littering intestines of several goats on ground) in Kotido district of Uganda, all in an attempt to ‘cure’ the disease. Such practices only enhanced the spread of the disease and complicated the economic cost of the outbreak [26, 27]. This also has implications for other highly infectious diseases such as Lassa fever and COVID-19 that require isolation of confirmed positive cases as part of containment. In such situations, similar preference for alternative treatment options may negatively impact control efforts.

The factor that was found to significantly influence participants knowledge about EVD was their educational status. The agrarian community dwellers with at least secondary education in this study were more likely to have good knowledge of EVD compared to those with only primary or no formal education. This highlights the need to increase education coverage in local communities as the level of education of the populace could play an important role in determining the magnitude of spread as modelled by outcomes in two separate outbreaks in Sudan [28].

Most respondents indicated stigmatizing attitudes towards EVD survivors. A total of 40% stated that they will not buy any goods from a survivor, many expressed that they will not welcome a survivor back into the community nor allow survivor into their house. These discriminatory statements were similar to the initial problems local residents at Ebola epicenters posed during early phases of the 2014 outbreak in Liberia [29]. The danger in this is that persons that suspect that they may have EVD, and indeed any infectious disease hide it because of fear of stigmatization. This could drive disease outbreaks further.

During the outbreak, the preventive method most respondents in this study observed was avoiding bush meat and use of salt water which are largely misconceptions. The use of salt water may have negative health consequences. Though the exposure is there with consumption of bush meat, the key thing is close contact and method of handling during preparation of the animals. This was not really a big issue in EVD outbreak in Nigeria as the cases recorded were invariably linked to the imported case. The natives already exhibited poor knowledge and bush meat is commonly consumed due to their agro-forestry background hence the need for proper education. One year later, majority of the discussants stated that they had resumed bush meat consumption and were no longer taking any recommended precautions to prevent contracting EVD. The main reason for this in-action could be linked to their religious belief, that ‘God’ protects them from ‘evil diseases’ like EVD (Table 8). Unfortunately, this behavior may have serious consequences in the re-occurrence of EVD outbreak in the country.

**Table 8:** Explanatory models of Ebola Virus Disease from FGDs

EVD domains	HS group	OF group	AM group	RF group
**Cause**	Germ	Punishment for sins	Greed	Germ
**Transmission**	Contact with infected body fluids/ animals	Eating bush meat	Sexual intercourse	Eating bush meat, contact with infected body fluids
**Treatment**	Medical hospital, local herbalist,	*no recurrent theme*	Local herbalist, medical hospital	Local herbalist
**Burial of victims**	To be done by healthcare personnel	Opposed to cremation	No to burial rites but opposed to cremation	To be done by government. Opposed to cremation
**Prevention during outbreak**	Stopped eating bush meat, used salt water	Salt water	Salt water	Salt water
**Prevention post-outbreak**	None. Resumed eating bush meat	None. ‘God’ protects	Protected intercourse	None. ‘God’ protects

### Strengths and limitations

The study was conducted in a setting that can be described as ‘high risk’ for EVD outbreak. Data was collected prospectively, and the mixed-method approach yielded more information necessary for understanding community explanatory models of the disease in the context of outbreak preparedness and control.

The study did not emphasize on how local beliefs and practices could aid control efforts in such epidemics. More content could have been covered by adapting Dunn’s framework [30] and this could be addressed in larger scale studies. The grading system adopted for measuring ‘attitude’ could have affected the result of the overall attitude (majority had good attitude) as their ‘neutrality’ was not factored into the grading system. No case of EVD was recorded in the study area during the outbreak, nevertheless the limited data provides relevant information useful to researchers and other public health stakeholders in infectious disease prevention and control.

## Conclusion

The study has shown very poor knowledge of EVD with misconceptions. Though majority perceived the disease to be severe, some believed it was a ploy of whites against African Countries and avenue for government officials to embezzle money. Respondents exhibited stigmatizing attitude which may hinder control efforts in disease outbreaks. They were also not pro-active about prevention of possible future outbreak as most had gone back to harmful practices initially abandoned because the outbreak was declared to be over in the country.

## Data Availability

The datasets used and/or analyzed during the study are available from the corresponding author on reasonable request.

## Abbreviations

EVD: Ebola Virus Disease
RNA: Ribonucleic acid
DRC: Democratic Republic of Congo
COVID: Corona Virus Disease
FGD: Focus Group Discussion
WHO: World Health Organization
HS: Higher secondary School students
AM: Adult males
OF: Older females
RF: Women of reproductive age
